# CDK-dependent phosphorylation of PHD1 on serine 130 alters its substrate preference in cells

**DOI:** 10.1242/jcs.179911

**Published:** 2016-01-01

**Authors:** Brian Ortmann, Dalila Bensaddek, Sara Carvalhal, Sandra C. Moser, Sharon Mudie, Eric R. Griffis, Jason R. Swedlow, Angus I. Lamond, Sonia Rocha

**Affiliations:** Centre for Gene Regulation and Expression, School of Life Sciences, University of Dundee, Dow Street, Dundee DD1 5EH, UK

**Keywords:** EGLN2, HIF, Cep192, Hypoxia

## Abstract

PHD1 (also known as EGLN2) belongs to a family of prolyl hydroxylases (PHDs) that are involved in the control of the cellular response to hypoxia. PHD1 is also able to regulate mitotic progression through the regulation of the crucial centrosomal protein Cep192, establishing a link between the oxygen-sensing and the cell cycle machinery. Here, we demonstrate that PHD1 is phosphorylated by CDK2, CDK4 and CDK6 at S130. This phosphorylation fluctuates with the cell cycle and can be induced through oncogenic activation. Functionally, PHD1 phosphorylation leads to increased induction of hypoxia-inducible factor (HIF) protein levels and activity during hypoxia. PHD1 phosphorylation does not alter its intrinsic enzymatic activity, but instead decreases the interaction between PHD1 and HIF1α. Interestingly, although phosphorylation of PHD1 at S130 lowers its activity towards HIF1α, this modification increases the activity of PHD1 towards Cep192. These results establish a mechanism by which cell cycle mediators, such as CDKs, temporally control the activity of PHD1, directly altering the regulation of HIF1α and Cep192.

## Abstract (precis)

**Summary:** CDK-mediated phosphorylation of PHD1 at serine 130 controls target specificity and confers cell cycle regulation of PHD1.

## INTRODUCTION

Decreased oxygen levels, or hypoxia, present a major stress to the cell. Many of the crucial cellular processes, such as ATP production through oxidative phosphorylation, cell division and cell cycle progression are all highly energy-demanding processes that require oxygen (Ortmann et al., 2014). Exposure to hypoxia activates a number of different responses at both the cellular and whole organism level. One of the crucial alterations mediated by hypoxia is a change in gene expression (Kenneth and Rocha, 2008).

Hypoxia activates a variety of transcription factors (Kenneth and Rocha, 2008), but the most important for survival and adaptation to this stress is a group of transcription factors known as the hypoxia-inducible factors (HIFs). These are heterodimeric transcription factors that comprise an oxygen-labile HIFα subunit and the constitutively expressed HIF1β subunit (also known as ARNT) (Moniz et al., 2014). Three different genes encode for the currently known isoforms of the HIFα subunit [HIF1α, HIF2a (also known as EPAS1) and HIF3α]. All of the three HIFα isoforms share some structural similarity, most notably they all contain a basic helix-loop-helix Per-Arnt-Sim (bHLH-PAS) domain, which is crucial for its interaction with its transcriptional partner HIF1β (To et al., 2006). In addition, they also contain an oxygen-dependent degradation domain (ODD), which renders these proteins sensitive to proteosomal degradation in the presence of oxygen. Although transcription and translation of the HIFα isoforms plays a role in the control of these transcription factors (Bernardi et al., 2006; Nayak et al., 2013; van Uden et al., 2008, 2011), the oxygen-dependent control of HIFα is achieved through protein degradation, which occurs very rapidly in the presence of oxygen (Fandrey et al., 2006).

During normoxia, when cells have access to oxygen, HIFα is hydroxylated on two key proline residues located within the ODD domain by a group of proline hydroxylase enzymes (PHDs). PHDs require molecular oxygen as a co-substrate to carry out hydroxylation, but they also have a requirement for α-ketoglutarate (α-KG) and Fe^2+^ as cofactors (Fandrey et al., 2006). As α-KG is a key component of the Krebs cycle, it is thought that, in addition to sensing oxygen levels, PHDs can also sense the metabolic state within the cell (Kaelin, 2012). More recent data have shown that the PHDs are also important for amino acid sensing (Durán et al., 2013). Currently there are three known isoforms in mammalian cells (PHD1, PHD2 and PHD3, also known as EGLN2, EGLN1 and EGLN3, respectively), all of which have the ability to hydroxylate HIFs. HIF1α is hydroxylated on P402 and P564, whereas HIF2α is hydroxylated on P405 and P531. Biochemical analysis of all three isoforms has shown that PHD2 has the highest affinity for HIFs, but, interestingly, the PHDs also possess preferential affinities for the proline that they target (Appelhoff et al., 2004; Berra et al., 2003). Genetic studies have shown that, out of the three isoforms, deletion of PHD2 is embryonic lethal (Takeda et al., 2006), whereas deletion of PHD1 and PHD3 are not. However, loss of PHD1 and/or PHD3 lead to developmental defects, most notably in the cardiovascular system (Fong and Takeda, 2008; Takeda et al., 2008).

During normoxia, hydroxylation of HIFα creates a binding site for the von Hippel-Lindau (VHL) tumor suppressor E3 ligase complex. Binding of VHL results in polyubiquitylation and proteosomal degradation of HIFα. During hypoxia, when oxygen levels are decreased, PHD activity is reduced, leading to stabilization of HIFα and dimerization with HIF1β, resulting in a transcriptionally active complex. HIFs have been shown to regulate a large number of genes involved in a variety of cellular processes, such as metabolism, apoptosis, autophagy, angiogenesis and cell proliferation (Moniz et al., 2014; Rocha, 2007). The response engaged during hypoxia promotes cell survival and turns off highly energy-consuming processes, such as cell proliferation and translation.

One of the most energy-consuming processes within the cell is the cell cycle and, hence, cell division. This process must be tightly regulated to ensure there is no hyperproliferation and/or mis-segregation of genetic information. Errors within the cell cycle can ultimately lead to disease states, such as cancer. Cell cycle control is achieved through multiple mechanisms, but amongst the most important regulators are cyclin-dependent kinases (CDKs) (Besson et al., 2008; Bloom and Cross, 2007; Obaya and Sedivy, 2002). CDKs are a family of serine/threonine kinases, which are activated when the cell chooses to enter the cell cycle. Their activation is dependent on multiple factors, but most important is their interaction with their regulatory cyclins (Bloom and Cross, 2007). In addition, CDK activity is also regulated through their interaction with inhibitory proteins, such as p21 (also known as CDKN1A) and p27 (also known as CDKN1B). These proteins interact directly with the CDKs and inhibit the interaction with their regulatory cyclin (Besson et al., 2008).

Several studies have shown that hypoxia also affects the cell cycle. Early work has demonstrated that, upon exposure to hypoxia, cells are reversibly arrested in G1 or S phase (Gardner et al., 2001; Ortmann et al., 2014). The mechanisms controlling this arrest have been shown to be both HIF dependent and independent (Ortmann et al., 2014). More recent studies have shown that HIF1α can inhibit DNA replication independently of its transcriptional activity (Hubbi et al., 2013).

In more recent years, the roles of the PHDs in processes other than hypoxia have become more apparent. Recently PHD1 has been shown to regulate the transcription factor FOXO3A (Zheng et al., 2014). PHD3 has also been shown to hydroxylate PKM2 and HCLK2 (Luo et al., 2011; Xie et al., 2012). Moreover, our recent work has shown that PHD1 can regulate mitotic progression through its ability to control the levels of the key centrosomal component Cep192 (Moser et al., 2013). However, the mechanism that determines whether PHD1 targets Cep192, HIFα, or both, is not known. In addition, there is no information on how these enzymes engage with the cell cycle.

Here, we show that PHD1 is regulated by the cell cycle at the post-translational level. We show that PHD1 is phosphorylated at S130 in a CDK-dependent manner. PHD1 phosphorylation reduces its interaction with HIF1α, but increases the association between PHD1 with Cep192. Functionally, this results in increased levels of HIF1α protein and increased transcriptional activity in response to hypoxia, and in reduced levels of Cep192 protein. These results indicate that the behaviour of PHD1 towards different substrates can be altered by specific post-translational modifications.

## RESULTS

### PHD1 is phosphorylated on S130

**Fig. 1.**
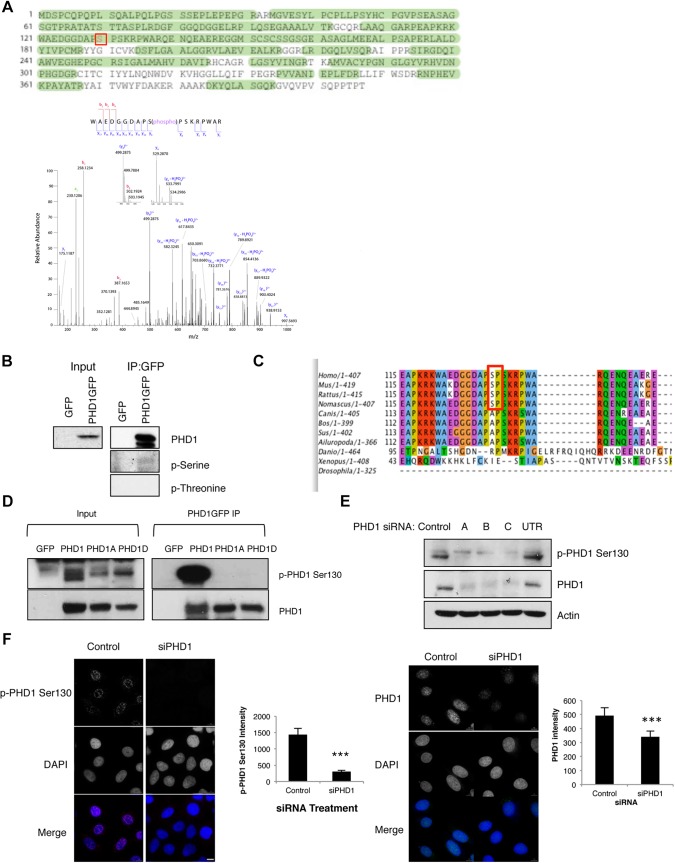
**PHD1 is phosphorylated at S130.** (A) LC-MS analysis of in-gel-digested PHD1 allowed the identification of PHD1 (UniProt Q96KS0) with 74.2% sequence coverage (searched against the UniProt human proteome database). The phosphorylated serine is denoted in red. ES-FTMS product ion spectrum of the triply charged ion at *m*/*z* value 688.3092, which corresponds to the tryptic peptide WAEDGGDAPS(phospho)PSKRPWAR. An almost complete y ion series allowed the unambiguous assignment of phosphorylation to S130 of PHD1. Higher y ions (from y_9_ to y_17_) are observed both with a phosphate group and with a loss of H_3_PO_4_. (B) 300 µg of U2OS GFP and PHD1–GFP cell extracts were subjected to immunoprecipitation (IP) using GFP-trap beads, and precipitated material was analysed by western blotting using the indicated antibodies. (C) Sequence alignment diagram for the PHD1 S130 site within different organisms. The box indicates conserved SP residues. (D) 300 µg of U2OS GFP, PHD1–GFP, PHD1-S130A–GFP and PHD1-S130D–GFP cell extracts were subjected to immunoprecipitation using GFP-trap beads, and precipitated material was analysed by western blotting using the indicated antibodies. (E) U2OS PHD1–GFP cells were transfected with control or several PHD1 siRNA oligonucleotides (denoted A, B, C and UTR) for 48 h prior to lysis. Whole-cell lysates were analysed by western blotting using the depicted antibodies. UTR, untranslated region. (F) U2OS cells were transfected with control or PHD1 siRNA (siPHD1) oligonucleotides for 48 h prior to fixation with PFA. Cells were stained with anti-phospho-S130 PHD1 and PHD1 antibodies, using DAPI as a marker of chromatin. Scale bar: 10 µm. Images were acquired using a Deltavision microscope, deconvolved and analysed using Omero software. Pixel intensities were quantified in Omero using the region of interest (ROI) tool. Graphs depict mean±s.d. of a minimum of 31 cells per condition. *P<0.05, **P<0.01, ***P<0.001 compared to control conditions (Student's *t*-test). p, phosphorylated form of the protein or residue. See also Fig. S1.

We performed mass spectrometry analysis to map PHD1 phosphorylation events by using extracts from U2OS cells expressing GFP-tagged PHD1. PHD1–GFP was immunoprecipitated from cells and then subjected to mass spectrometry analysis (Fig. 1A, Table 1). A good coverage of PHD1 peptides was obtained, and we found that S130 was phosphorylated in interphase cells (Fig. 1A). Mass spectrometry validation was achieved using antibodies that specifically recognise phosphorylated (phospho-)serine or threonine residues (Fig. 1B), confirming that PHD1 can be phosphorylated on serine residues but not on threonine residues (Fig. 1B). Sequence alignment of PHD1 from different organisms demonstrates that the S130 phosphorylation site in humans is highly conserved in higher mammals, mice and rats but is absent in organisms such as zebrafish, *Xenopus* and the fruit fly (Fig. 1C). An antibody against a synthetic phospho-peptide corresponding to the region around S130 was generated. Antibody specificity and validation was performed using U2OS cell lines stably expressing GFP, PHD1–GFP and two GFP-tagged PHD1 mutants cell lines where the S130 residue has been replaced with either an alanine (PHD1-S130A) or an aspartate (PHD1-S130D). All of these cells expressed PHD1 to similar levels (Fig. S1A). Immunoprecipitation of GFP from the GFP, PHD1–GFP, PHD1-S130A–GFP or PHD1-S130D–GFP cells, revealed that the phospho-specific antibody only detected a band in the extracts derived from wild-type PHD1, demonstrating its specificity (Fig. 1D). In addition, we knocked down PHD1 levels using several different small interfering RNA (siRNA) oligonucleotides directed against PHD1, and used the antibody to determine its specificity in cell extracts (Fig. 1E). A substantial loss of signal was detected specifically when PHD1 was depleted, further demonstrating the specificity of this antibody (Fig. 1E).

**Table 1.**
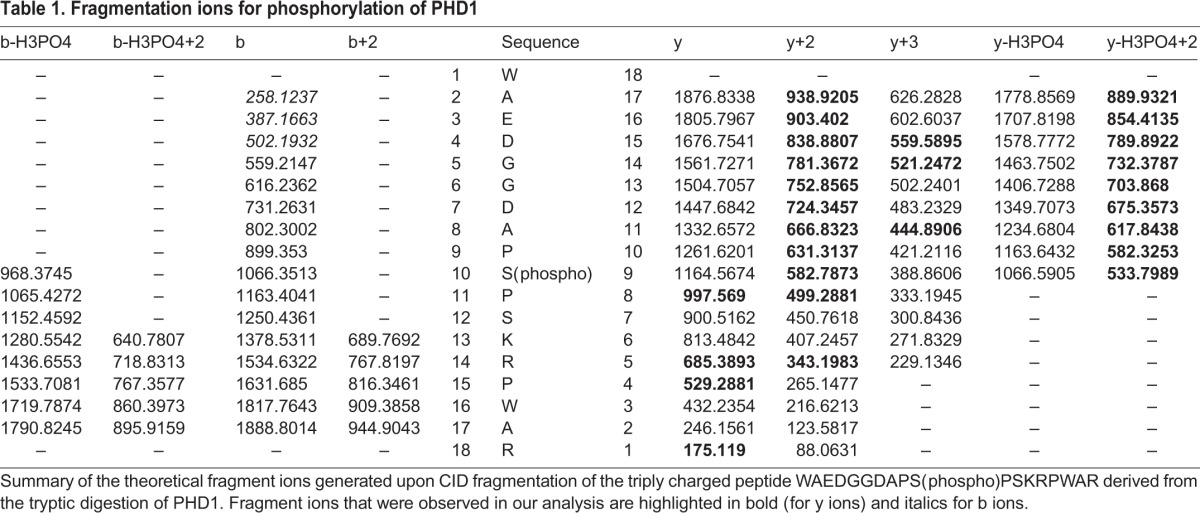
**Fragmentation ions for phosphorylation of PHD1**

Although, endogenous PHD1 levels are difficult to detect in cell lines other than breast cancer lines (Moser et al., 2013; Zheng et al., 2014), we used an immunofluorescence approach in U2OS cells to determine whether the antibody against phospho-S130 PHD1 was able to detect endogenous protein (Fig. 1F). PHD1 phosphorylation was localised primarily within the nucleus, consistent with previous data showing that PHD1 is a nuclear protein (Metzen et al., 2003). The PHD1 phosphorylation signal was lost when cells were treated with a PHD1-specific siRNA. The same localisation pattern was also observed in PHD1–GFP cells (Fig. S1B). Collectively, these results show that PHD1 is subject to phosphorylation on S130 in cells.

### PHD1 phosphorylation is regulated by interphase CDKs

**Fig. 2.**
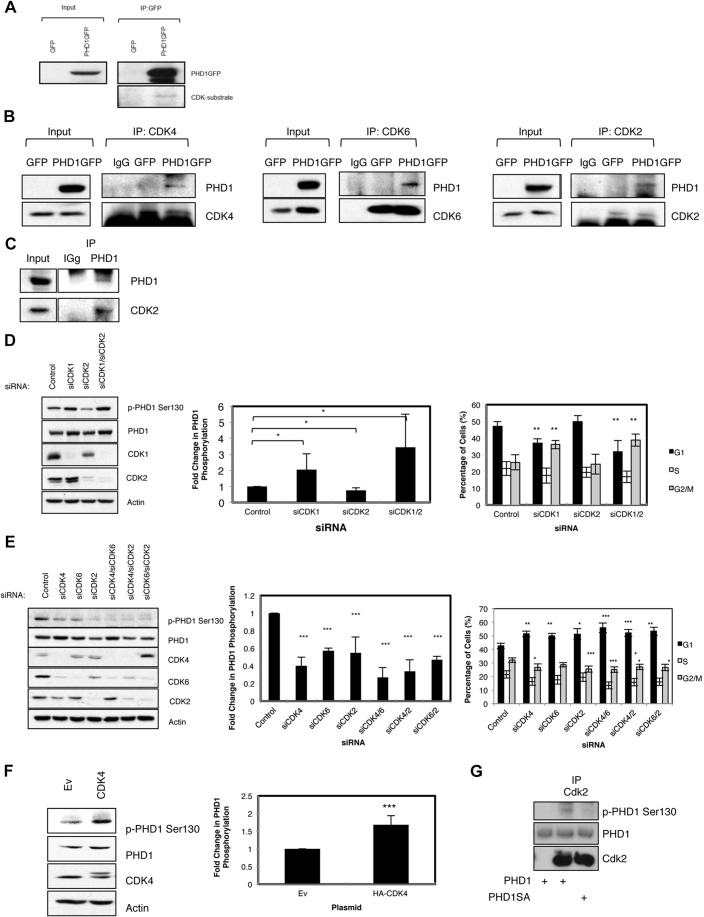
**PHD1 phosphorylation at S130 is regulated by CDKs.** (A) 300 µg of U2OS GFP or PHD1–GFP cell extracts were subjected to immunoprecipitation (IP) using GFP-trap beads, and precipitated material was analysed by western blotting using the indicated antibodies. (B) 300 µg of U2OS GFP or PHD1–GFP cell extracts were subjected to immunoprecipitation using antibodies towards CDK4, CDK6 and CDK2, and precipitated material was analysed by western blotting for the presence of PHD1. (C) 500 µg of U2OS cell extracts were subjected to immunoprecipitation using an anti-PHD1 antibody crosslinked to Sepharose beads and processed as in A. (D) U2OS PHD1–GFP cells were transfected with control, CDK1 or CDK2 siRNA (siCDK) oligonucleotides alone or in combination for 48 h prior to lysis for western blotting or fixation for FACS analysis. Cell lysates were analysed for the levels of phosphorylated PHD1 at S130 and appropriate controls. The middle graph depicts the mean±s.d. of the quantification of the western blot analysis, representing a minimum of three independent experiments. The right panel depicts the corresponding cell cycle profile of cells treated as mentioned (mean±s.d. of a minimum of three independent experiments). (E) U2OS PHD1–GFP cells were transfected with control or the indicated CDKs siRNAs alone or in combination for a period of 48 h prior to being processed and analysed as in D. (F) U2OS PHD1–GFP cells were transfected with 1 µg of control or CDK4 expression constructs for 48 h prior to lysis and analysed by western blotting for the levels of phosphorylated PHD1 at S130 and appropriate controls. Ev, empty vector control. Graph depicts mean±s.d. of the quantification of the western blot analysis, from a minimum of three independent experiments. (G) CDK2 was immunoprecipitated from cells and used in a kinase assay with 2 µg of recombinant PHD1 and PHD1-S130A protein. Reactions were analysed by western blotting with the indicated antibodies. *P<0.05, **P<0.01 and ***P<0.001 compared to control conditions (Student's *t*-test). p, phosphorylated form of the protein. See also Fig. S1.

Further analysis of the sequence surrounding the phosphorylation site revealed it falls into a consensus for CDK phosphorylation [S/T]Px[R/K] (Endicott et al., 1999). To investigate whether CDKs can modify PHD1, we determined whether PHD1 could be detected by an antibody specifically directed towards phospho-CDK substrates. PHD1–GFP was immunoprecipitated from cells, and extracts were probed with both the antibody for the CDK substrates and with an anti-PHD1 antibody (Fig. 2A). This revealed that a small proportion of PHD1 was indeed recognised by the anti-CDK-substrate antibody, highlighting the potential for PHD1 to be a CDK substrate. In addition, PHD1 could interact with CDK2, CDK4 and CDK6, but not CDK1 in cells (Fig. 2B; Fig. S1C). We could also detect an interaction between PHD1 and CDK2 at the endogenous level (Fig. 2C).

Functionally, depletion of CDK2, CDK4 and CDK6, either individually or in combination, resulted in reduced levels of phospho-S130 PHD1 (Fig. 2D,E). CDK1 depletion led to increased levels of this phosphorylation on PHD1 (Fig. 2D), with a corresponding increase in cells arrested in the G2 and M phase of the cell cycle. However, CDK depletion did not alter PHD1 localisation in cells (Fig. S1D). By contrast, gain-of-function experiments revealed that when increased levels of CDK4 were present, there was a concomitant increase in the levels of S130 phosphorylation of PHD1 (Fig. 2F), indicating that indeed CDKs can change the PHD1 phosphorylation status.

To demonstrate that CDKs can phosphorylate PHD1, we also performed *in vitro* kinase assays with CDK2 and CDK1 (Fig. 2G, Fig. S1E). CDK2 was immunoprecipitated from cells, and kinase assays were performed using bacterially expressed recombinant PHD1, followed by western blot analysis using the anti-phospho-PHD1 antibody (Fig. 2G). This analysis showed that CDK2 phosphorylates PHD1 at S130. Interestingly, when a radioactive kinase assay was performed with recombinant CDK1–cyclin-B, a CDK for which we were unable to detect an interaction with PHD1, we could detect phosphorylation of PHD1 *in vitro* (Fig. S1E). In this case, mutation of S130 only slightly reduced the phosphorylation signal, suggesting that other sites on PHD1 are being targeted by CDK1 *in vitro*. Taken together, these results suggest that no single CDK is exclusively responsible for the phosphorylation of PHD1, indicating a redundancy between them. This is to be expected, as genetic studies have shown that only CDK1 is essential, as knockout of CDK1 results in significant developmental defects in mice (Diril et al., 2012).

### S130 phosphorylation of PHD1 is regulated by the cell cycle and upregulated by oncogenes

**Fig. 3.**
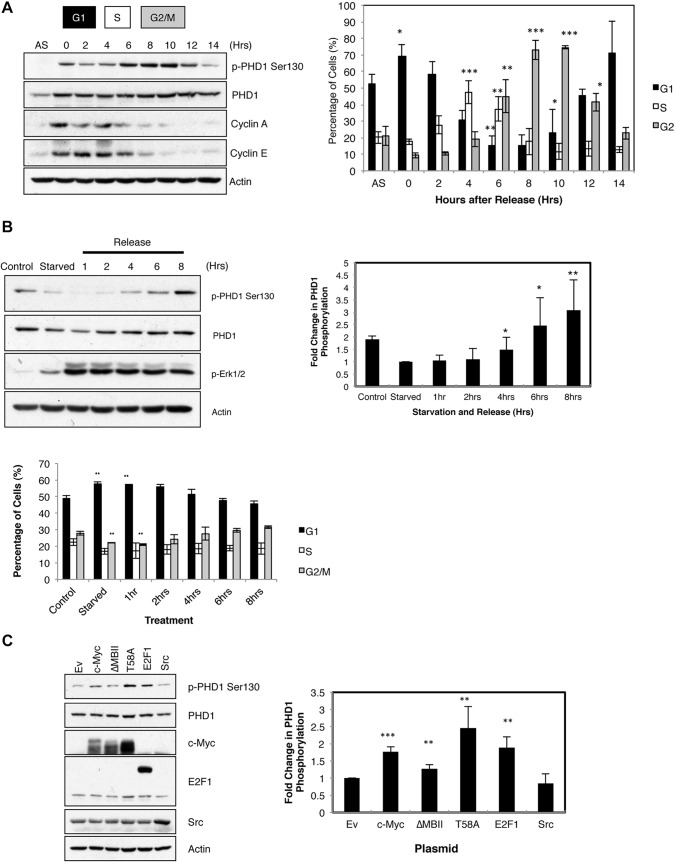
**PHD1 phosphorylation at S130 is regulated by the cell cycle and responds to mitogenic signalling.** (A) U2OS PHD1–GFP cells were subject to a double-thymidine block release protocol prior to lysis or fixation after the indicated periods of time. The left panel depicts western blot analysis for the levels of phosphorylated PHD1 at S130, and appropriate controls. The right panel represents the cell cycle profile of matching samples analysed by flow cytometry. AS, asynchronous. Graph depicts mean±s.d. of a minimum of three independent experiments. G1, S, and G2/M are the phases of the cell cycle that correspond to the indicated time points. (B) U2OS PHD1–GFP cells were serum starved for 24 h prior to addition of full serum medium and were harvested at the indicated times. Cells were lysed for western blot analysis or fixed for FACS analysis. Cell lysates were analysed for the levels of phosphorylated PHD1 at S130, and appropriate controls, where phosphorylated ERK1/2 was used as a marker of mitogenic signalling. The right graph depicts the mean±s.d. of the quantification of the western blot analysis, representing a minimum of three independent experiments. The bottom panel depicts the corresponding cell cycle profile of cells treated as mentioned above. The graph depicts the mean±s.d. of a minimum of three independent experiments. (C) U2OS PHD1–GFP cells were transfected with 1 µg of empty vector (Ev) control or the indicated oncogenes for 48 h prior to lysis for western blot analysis. Cell lysates were analysed for the levels of phosphorylated PHD1 at S130 and appropriate controls. The right graph depicts the mean±s.d. of the quantification of the western blot analysis, representing a minimum of three independent experiments. *P<0.05, **P<0.01 and ***P<0.001 compared to control conditions (Student's *t*-test). p, phosphorylated form of the protein.

Given that our results suggest that S130 phosphorylation is modulated by several CDKs, we hypothesised that this phosphorylation might be regulated by the cell cycle. Double-thymidine block followed by release over a 14-h period revealed that it was possible to detect S130 phosphorylation of PHD1 throughout the cell cycle. However, phosphorylation levels peaked during late S phase and G2 before decreasing again as the cell entered G1 (Fig. 3A). These results indicate that PHD1 phosphorylation is a cell-cycle-regulated event.

Growth factors are known to be one of the primary drivers for cell cycle progression, so we first tested whether S130 phosphorylation was regulated by growth factor stimulation (Fig. 3B). After starving the cells for 24 h, S130 phosphorylation was decreased when compared with the control, and this was coupled with an increase in the percentage of cells in the G1 phase of the cell cycle (Fig. 3B). Following release, there was a gradual increase in PHD1 phosphorylation, which coincided with an increase of cells moving into S and G2 phases of the cell cycle. Interestingly, this increase was delayed when compared with ERK1 and ERK2 (ERK1/2, also known as MAPK3 and MAPK1, respectively) phosphorylation, indicating that the kinase responsible is activated later than the ERK pathway.

A primary driver of cell proliferation and cell cycle progression in the context of cancer is the activation of oncogenes (Matsumura et al., 2003). We overexpressed the oncogene Myc and two mutant derivatives. One of the mutants contains a deletion in the Myc box II domain (ΔMBII), which prevents full transactivation of Myc (Cowling and Cole, 2008). The other mutant has a T58A mutation and acts as a Myc gain-of-function mutant (Welcker et al., 2004). T58 is a known GSK3 phosphorylation site and, without this site, Myc has increased stability, making it more active (Welcker et al., 2004). We also tested the effects of overexpression of E2F1 and Src. Overexpression of either wild-type Myc or either of the mutants, led to an increase in PHD1 phosphorylation, although the increase in PHD1 phosphorylation when overexpressing ΔMBII was not very pronounced (Fig. 3C, lanes 2–4). PHD1 phosphorylation also increased when E2F1 was overexpressed (Fig. 3C, lane 5), but not when we overexpressed Src (Fig. 3C, lane 6). This shows that S130 phosphorylation of PHD1 can be induced by increased levels of oncogenes, such as Myc and E2F1.

### PHD1 phosphorylation regulates HIFα levels and activity

**Fig. 4.**
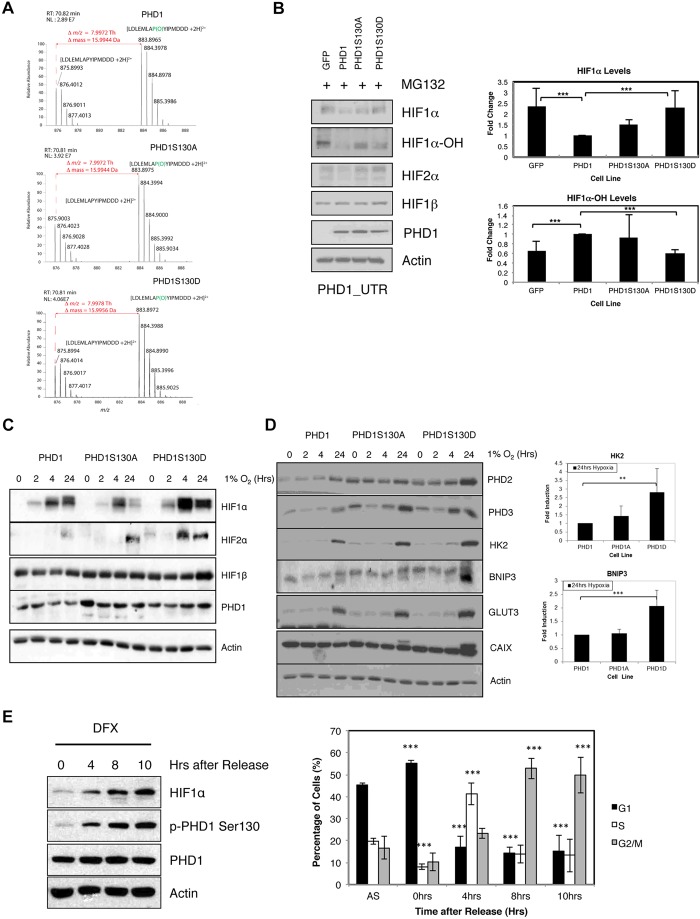
**PHD1 phosphorylation at S130 modulates HIF mediated responses to hypoxia.** (A) Recombinant purified PHD1, PHD1-S130A and PHD1-S130D enzymes were used in an *in vitro* hydroxylation assay using a peptide derived from the HIF1α ODD region (containing proline 564). Reactions were stopped with the addition of DFX, and samples were analysed by mass spectrometry. Electrospray-MS spectrum of the product of *in vitro* hydroxylation of the HIF1α peptide LDLEMLAPYIPMDDD showing an *m/z* increment of 7.99 Th (mass increment of 15.9944 Da) corresponding to proline hydroxylation of the doubly charged ion at *m/z* 875.8993 Th and the formation of the ion 883.8965 Th (the mass of the hydroxylated peptide) corresponding to the hydroxylation of proline 564 of HIF1α. The ion normalized level (NL) for the hydroxylated peptide is 2.89×10^7^ for wild-type PHD1, 3.92×10^7^ for PHD1-S130A and 4.06×10^7^ for PHD1-S130D. (B) U2OS GFP, PHD1–GFP, PHD1-S130A–GFP and PHD1-S130D–GFP were transfected with PHD1 siRNA targeting the 3′-UTR (untranslated region) of endogenous PHD1 mRNA for 48 h prior to treatment with MG132 for 3 h. Whole-cell lysates were analysed by western blotting for the levels of the indicated proteins. Graph depicts western blot quantification showing mean±s.d. of a minimum of three independent experiments. (C) U2OS PHD1–GFP, PHD1-S130A–GFP and PHD1-S130D–GFP cells were exposed to 1% O_2_ for the indicated periods of time prior to lysis. Whole-cell lysates were analysed by western blotting using the indicated antibodies. (D) Cell extracts obtained in C were analysed for the levels of the indicated HIF-dependent targets by western blotting. The graph depicts the quantification of western blots for HK2 and BNIP3, and illustrates the mean±s.d. for a minimum of three independent experiments. (E) U2OS PHD1–GFP cells were subject to a double-thymidine block release protocol prior to lysis or fixation on the indicated periods of time. For the last 3 h of each time point, 200 µM DFX was added to the cells. The left panel depicts western blot analysis for the levels of phosphorylated PHD1 at S130 and appropriate controls. The right panel represents the cell cycle profile of matching samples analysed by flow cytometry. AS, asynchronous. The graph depicts mean±s.d. of a minimum of three independent experiments. *P<0.05, **P<0.01 and ***P<0.001, compared to control conditions or as indicated (Student's *t*-test). See also Fig. S2.

As PHD1 is a proline hydroxylase, we next determined whether phosphorylation of PHD1 at S130 could impact on its intrinsic enzymatic activity. Previous work performed in bacteria has shown that PHD1 is potentially phosphorylated on S132 (Li et al., 2008) and this can lead to a decrease in PHD1 activity *in vitro*. To analyse PHD1 activity we used an *in vitro* hydroxylation assay, using a peptide derived from the HIF1α ODD sequence, followed by mass spectrometry (Fig. 4A). This analysis revealed that all the mutants had similar activity to the wild-type enzyme *in vitro*. We also performed this analysis using dot blot and an anti-HIF1α-hydroxylation antibody (Fig. S2A). We compared hydroxylation of the HIF peptide over time using recombinant purified GST–PHD1, GST–PHD1-S1301A and GST–PHD1-S130D proteins. No significant difference in hydroxylation activity between the wild-type PHD1 and the two mutants could be detected (Fig. S2A). These data indicate that phosphorylation of PHD1 at S130 has little or no effect on the intrinsic enzymatic activity of PHD1.

To determine whether there is a functional role of PHD1 S130 phosphorylation in the cellular response to hypoxia, we started by analysing the levels of its targets, that is HIF1α and HIF2α. To this aim, we utilized the GFP, PHD1–GFP, PHD1-S130A–GFP and PHD1-S130D–GFP U2OS cells, and assessed their PHD activity by measuring both the levels of hydroxylated HIF1α and total levels of HIF1α and HIF2α when their degradation was blocked by a proteasomal inhibitor (Fig. 4B). Specificity of the anti-hydroxy-HIF1α antibody was confirmed by analysis of extracts from cells treated with the PHD inhibitors DFX and DMOG (Fig. S2B). In addition, endogenous PHD1 was depleted by siRNA and, as expected, when MG132 was added, we saw an accumulation of HIF1α in all cell types. However, there were decreased levels of HIF1α in the PHD1–GFP and PHD1-S130A–GFP cells, whereas HIF1α levels in PHD1-S130D–GFP cells were similar to the cells expressing GFP alone (Fig. 4B). Similarly, when we analysed HIF1α hydroxylation levels, less hydroxylation was detected in PHD1-S130D–GFP cells when compared with PHD1-S130A–GFP cells. This result suggests that phosphorylation of PHD1 on S130 does impact on the ability of PHD1 to hydroxylate HIF1α in cells. Interestingly, mutation of S130 alters the PHD1-mediated regulation of HIF2α, regardless of mutation to alanine or aspartate (Fig. 4B). As expected, none of the PHD1 mutations affected the total levels of HIF1β.

In cells exposed to hypoxia, PHD1 overexpression results in a reduction of HIF1α levels and its targets as expected (Fig. S2C,D). Hypoxia does not alter PHD1 phosphorylation at earlier times of exposure but does result in a significant reduction after 24 h (Fig. S2E). This is to be expected, as exposure to hypoxia for this period results in G1 arrest (Ortmann et al., 2014), a stage where PHD1 S130 phosphorylation is reduced. When all three cell lines were exposed to hypoxia, we detected an induction of HIF1α in all of them (Fig. 4C), although to lower levels than control cells (Fig. S2C,F). However, for the PHD1-overexpressing cells, the highest induction of HIF1α was observed in the PHD1-S130D–GFP cells (Fig. 4C). This highlights that the change in hydroxylation activity we observed when we treated cells with MG132 (Fig. 4B) is physiologically relevant. In addition, levels of HIF2α are higher in both of the PHD1 mutant cell lines, this being particularly evident in PHD1-S130D­–GFP cells (Fig. 4C). Despite being less efficient at targeting HIF1α, PHD1-S130D–GFP is active, as the levels of HIF1α in these cells are lower than cells expressing GFP alone (Fig. S2F), indicating that PHD1 hydroxylase activity is still present when S130 is phosphorylated.

To understand whether the changes in HIF1α levels observed in the PHD1-S130D–GFP cells are altering HIF1α transcriptional activity, we assessed HIF activity by investigating the levels of several HIF target genes. Levels of HIF1α targets were always higher in PHD1-S130D–GFP cells when compared with cells expressing wild-type PHD1. However, additional differences were also observed for PHD1-S130A–GFP cells when compared with cells expressing wild-type PHD1, with higher levels of PHD2, PHD3 and CAIX (also known as CA9) observed in these cells (Fig. 4D). This could reflect the contribution of HIF2α to the regulation of certain targets (Elvidge et al., 2006). We also analysed mRNA levels for BNIP3, CAIX and Glut3 (also known as SLC2A3), at 24 h following exposure to hypoxia in these cells (Fig. S2G). These results show that levels of HIF1α targets are always higher in PHD1-S130D–GFP cells when compared with cells expressing wild-type PHD1. In this analysis, PHD1-S130A–GFP activity was comparable to wild-type PHD1, for all the genes analysed (Fig. S2G).

We have shown that PHD1 S130 phosphorylation is regulated by the cell cycle. As such, we next determined whether cells where PHD1 phosphorylation is high would have different levels of HIF1α. To this end, we synchronised cells with a double-thymidine block, and then released them into fresh medium containing the PHD inhibitor DFX, and then visualised HIF1α. We initially determined whether PHDs were still active under these conditions by investigating the levels of hydroxylated HIF1α in a time course of DFX treatment (Fig. S2H). We thus chose a 3-h DFX treatment because, at this time point, HIF is stabilised but still hydroxylated, and hence PHD activity changes could still be monitored. When we investigated HIF1α levels in the different stages of the cell cycle, we could observe that cells in G1 had lower levels of PHD1 S130 phosphorylation as well as lower HIF1α levels (Fig. 4E). By contrast, when cells were synchronised in either S, or G2 and M phase, PHD1 S130 phosphorylation increased and so did HIF1α levels (Fig. 4E). Taken together, this analysis reveals that both the phosphorylation-mimicking mutation S130D and increased S130 phosphorylation results in reduced PHD1 activity towards HIF1α, leading to both increased HIF1α levels and increased activity of this transcription factor under hypoxia.

### PHD1 phosphorylation results in increased HIFα half-life by reducing PHD1–HIFα interaction

**Fig. 5.**
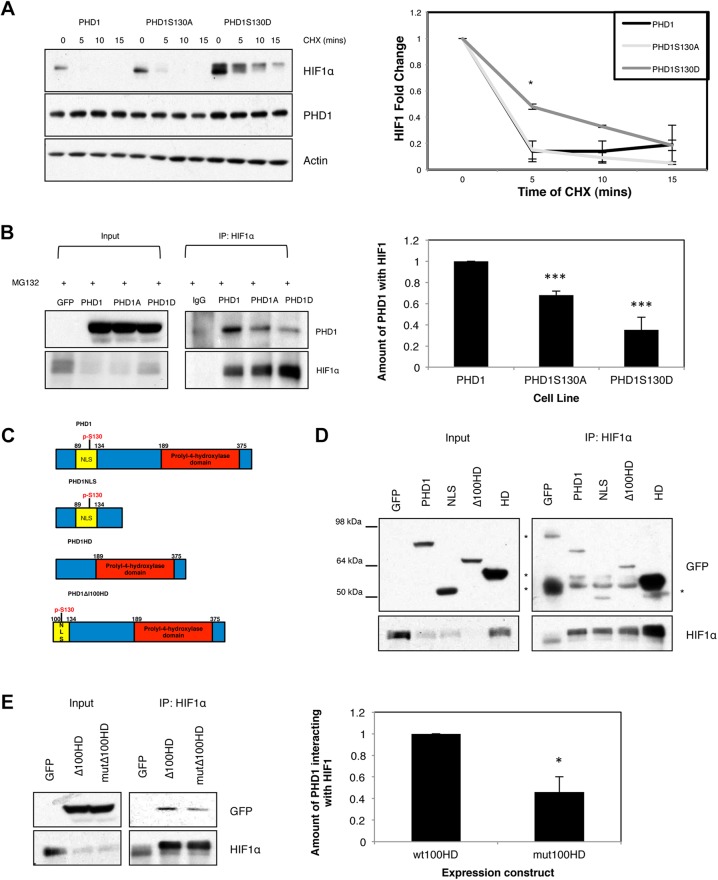
**PHD1 phosphorylation at Serine 130 alters the ability of PHD1 to target HIF1α.** (A) U2OS PHD1–GFP, PHD1-S130A–GFP and PHD1-S130D–GFP cells were exposed to 1% O_2_ for 4 h prior to treatment with cycloheximide for the indicated periods of time. Whole-cell lysates were analysed by western blotting for the levels of HIF1α and appropriate controls. Western blots were quantified and the graph depicts mean±s.d. of a minimum of three independent experiments. (B) U2OS GFP, PHD1–GFP, PHD1-S130A–GFP and PHD1-S130D–GFP cells were treated with MG132 for 3 h prior to lysis. 300 µg of cell extracts were used to immunoprecipitate (IP) HIF1α, with normal mouse IgG used as a control. Precipitated material was analysed by western blotting for the indicated proteins. Western blots were quantified, and the graph depicts the mean±s.d. of a minimum of three independent experiments. (C) Schematic diagram of the PHD1 expression constructs used in this study. Highlighted are the nuclear localization signal (NLS), S130 and the hydroxylase domain (HD). (D) HEK293 cells were transfected with 1 µg of the indicated expression constructs for 48 h prior to treatment with MG132 and processed as in B. *, non specific band. (E) HEK293 were transfected with 1 µg of the indicated expression constructs for 48 h prior to treatment with MG132 and processed as in B. Western blots were quantified, and the graph depicts mean±s.d. of a minimum of three independent experiments. *P<0.05, **P<0.01 and ***P<0.001 compared to control conditions (Student's *t*-test). See also Fig. S3.

Our analysis so far has revealed that PHD1 phosphorylation does not alter intrinsic enzymatic activity *in vitro* but does so in the context of cells, leading to increased levels of HIF1α levels and activity. To understand the mechanism behind these differences, we started by measuring HIF1α half-life in GFP, PHD1–GFP, PHD1-S130A–GFP and PHD1-S130D–GFP cells, using a cycloheximide chase approach (Fig. 5A; Fig. S3A). Cells were incubated under hypoxia for 4 h prior to treatment with cycloheximide for the indicated periods of time. We measured p53 levels as a positive control for the treatment, as it is known to have a high turnover rate (Fig. S3A). PHD1 levels were stable throughout the timecourse of the experiment in all cell lines (Fig. 5A; Fig. S3A). Interestingly, although PHD1–GFP and PHD1-S130A–GFP cells led to a substantial decrease in HIF1α half-life when compared with GFP cells, PHD1-S130D–GFP cells had a slower rate of degradation of HIF1α (Fig. 5A; Fig. S3A). These results are in agreement with the increased HIF1α levels observed in PHD1-S130D–GFP cells when exposed to either MG132 or hypoxia.

To determine the mechanism behind the loss of PHD1 targeting of HIF1α in PHD1-S130D–GFP cells, we next investigated whether this modification alters the ability of PHD1 to interact with HIF1α in cells. To this end, we treated cells with MG132, to stabilise HIF1α, and immunoprecipitated endogenous HIF1α from all the PHD1 cell lines. Normal IgG was used as a negative control for this approach (Fig. 5B). As can be seen in the left panel of Fig. 5B, there was a substantial immunoprecipitation of HIF1α, with higher levels of HIF1α recovered from the PHD1-S130D–GFP cells. However, the amount of interacting PHD1 was reduced in these cells, when compared with PHD1–GFP cells (Fig. 5B). Interestingly, a reduction in the interaction between HIF1α and PHD1 was also visible in PHD1-S130A–GFP cells. We also analysed this interaction using a transient transfection approach in HEK293 cells (Fig. S3B), again using MG132 treatment to stabilise HIF1α prior to immunoprecipitation. In this system, we could again detect a reduction in the level of PHD1 that interacts with HIF1α in the cells transfected with the PHD1-S130D–GFP construct (Fig. S3B). By contrast, there was no difference between PHD1–GFP- and PHD1-S130A–GFP-transfected cells in the levels of interaction between PHD1 and HIF1α. These results suggest that phosphorylation of PHD1 at S130 disrupts the interaction between HIF1α and PHD1.

We next determined whether the N-terminal region of PHD1 can bind HIF1α. Different PHD1 deletions were created according to the domain structure for PHD1 (Fig. 5C) and the subcellular localisation of these mutants was assessed by fluorescence microscopy. This revealed that all deletion constructs had a nuclear localisation, with the exception of the construct containing the hydroxylase domain only, which was diffusely distributed throughout the cell (Fig. S3C). This is to be expected, as this construct lacks the nuclear localisation signal. Furthermore, we could detect good expression levels of all of the constructs in cells when these were analysed by western blotting (Fig. S3D). Interaction assays with the deletion constructs revealed that both the N-terminal and C-terminal regions of PHD1 were able to interact with HIF1α in cells (Fig. 5D). We also analysed the effect of a phospho-mimicking mutation on the construct containing the hydroxylation domain but lacking the first 100 amino acids. Under these conditions, although the levels of immunoprecipitated HIF1α were similar for both PHD1 constructs, we observed a reduced interaction with the PHD1 construct containing the S130D mutation (Fig. 5E). These results suggest that the N-terminal region of PHD1, and in particular S130, is important for PHD1 binding to HIF1α.

### PHD1 S130 is important for the control of the cell cycle

**Fig. 6.**
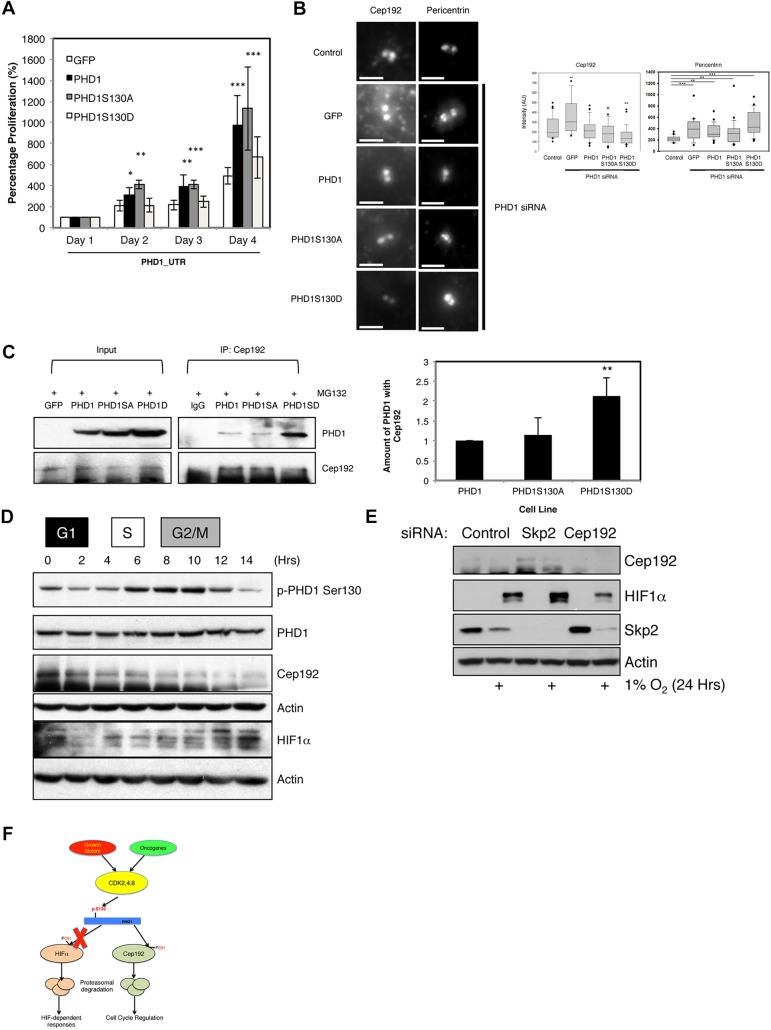
**S130 of PHD1 is important for PHD1-mediated control of cell proliferation.** (A) U2OS GFP, PHD1–GFP, PHD1-S130A–GFP and PHD1-S130D–GFP cells were transfected with siRNA oligonucleotides targeting the 3′UTR of endogenous PHD1 prior to proliferation being assessed. Total cell numbers were counted, and the graph depicts mean±s.d. of a minimum of three independent experiments. Data were normalised to proliferation in GFP cells and expressed as a percentage. (B) U2OS GFP, PHD1GFP, PHD1-S130A–GFP and PHD1-S130D–GFP cells were transfected with siRNA oligonucleotides targeting the 3′UTR of endogenous PHD1 prior to fixation and immunostaining for Cep192 and pericentrin. Scale bars: 2 µm. Graph depicts box-and-whisker plots for Cep192 and Pericentrin intensity. Box-and-whisker plot, middle line shows the median value; the bottom and top of the box show the lower and upper quartiles (25-75%); whiskers extend to 10th and 90th percentiles, and all outliers are shown. *n*=22–38 cells per condition. (C) U2OS GFP, PHD1GFP, PHD1-S130A–GFP and PHD1-S130D–GFP cells were treated with MG132 for 3 h prior to lysis. 300 µg of cell extracts were used to immunoprecipitate (IP) Cep192, with normal mouse IgG used as a control. Precipitated material was analysed by western blotting for the indicated proteins. Western blots were quantified and the graph depicts mean±s.d. of a minimum of three independent experiments. (D) Cell extracts from Fig. 3A were analysed by western blotting for the levels of Cep192 and HIF1α. G1, S, and G2/M are the phases of the cell cycle that correspond to the indicated time points. (E) U2OS were transfected with the indicated siRNAs prior to treatment with 1% O_2_ for 24 h. Whole-cell lysates were analysed by western blotting using the depicted antibodies. (F) Schematic diagram for the proposed model for PHD1 regulation by CDKs. *P<0.05, **P<0.01 and ***P<0.001 compared to control (Student's *t*-test). See also Fig. S4.

We previously identified Cep192 as a new PHD1 target (Moser et al., 2013). Cep192 is important for cell cycle progression, in particular for controlling the process of mitosis and spindle assembly (Joukov et al., 2014; Moser et al., 2013). We next determined the impact of PHD1 S130 on cell proliferation (Fig. 6A). In cells depleted of endogenous PHD1 and only expressing GFP, there was no evident cell proliferation. However, this was restored in cells expressing exogenous wild-type PHD1 and also in cells expressing PHD1 with the S130A mutation (Fig. 6A). Interestingly, PHD1-S130D was unable to restore proliferation in cells depleted of endogenous PHD1 (Fig. 6A). This suggests that phosphorylation of S130 in PHD1 alters PHD1 function, either towards Cep192 directly, or to an alternative, as yet unknown, substrate controlling cell cycle progression.

Given the defects observed in proliferation of cells expressing PHD1-S130D, we next investigated the levels of Cep192 in all PHD1 cell lines by performing immunofluorescence studies. Depletion of PHD1 resulted in an increase in Cep192 levels in GFP cells (Fig. 6B), as previously published (Moser et al., 2013). Expression of either wild-type PHD1, or the PHD1-S130A mutant, restored Cep192 levels to that seen in control siRNA GFP cells (Fig. 6B). Importantly, in PHD1-S130D–GFP cells, levels of Cep192 were significantly lower than in either control siRNA-depleted cells, or in PHD1–GFP and PHD1-S130A–GFP cells, indicating that the phospho-mimic mutant of PHD1 has increased activity towards Cep192 in cells (Fig. 6B). As Cep192 is important for the recruitment of centrosomal components, we also investigated the levels of pericentrin under the same conditions. Once again, GFP-expressing cells depleted of PHD1 had higher levels of pericentrin, as we have previously reported (Moser et al., 2013). Again, although PHD1 and PHD1-S130A rescued this effect, expression of PHD1-S130D, did not (Fig. 6B).

Given the reduced levels of Cep192 in PHD1-S130D cells, we hypothesised that once phosphorylated, PHD1 had enhanced activity towards Cep192. To investigate this, we analysed whether PHD1 could interact differently with Cep192 in wild-type versus PHD1 S130 mutant cells. This analysis revealed that increased levels of PHD1 were immunoprecipitated with Cep192 in phospho-mimicking PHD1 cells (Fig. 6C). By contrast, the non-phosphorylatable mutant PHD1 had a similar ability to interact with Cep192 as wild-type PHD1 (Fig. 6C). Once again, when we analysed the activity of recombinant PHD1 wild-type and mutants *in vitro*, using a peptide from Cep192 followed by mass spectrometry analysis, we could not detect any change in PHD1 hydroxylase activity (Fig. S4A), indicating that phosphorylation is modulating PHD1 activity only in cells, and not affecting PHD1 intrinsic hydroxylase activity.

To determine whether S130 phosphorylation correlated with Cep192 levels, we analysed the synchronisation release samples described in Fig. 3A for the levels of Cep192 (Fig. 6D). There, it was possible to observe that Cep192 levels were inversely correlated with PHD1 phosphorylation at S130 and HIF1α levels (Fig. 6D). Our results suggest that S130 phosphorylation of PHD1 results in reduced targeting of HIF1α and increased targeting of Cep192 by this enzyme. We next investigated whether alteration in the levels of Cep192 resulted in changes to the levels of HIF1α. To this end, we depleted either Cep192 or the ubiquitin ligase Skp2, which we have previously shown to regulate Cep192 levels in a manner dependent on PHD1 (Moser et al., 2013). Reducing the levels of Cep192 resulted in reduced levels of HIF1α. Conversely, in the absence of Skp2, when more Cep192 was present, higher levels of HIF1α were observed (Fig. 6E). Skp2 itself is regulated by hypoxia, due to the fact that cells arrest in G1, and Skp2 levels are regulated by the cell cycle (Ortmann et al., 2014; Yamada et al., 2013). Similar results were also observed in cells overexpressing Cep192 (Fig. S4B). These results suggest that competition between the HIF and Cep192 substrates can occur.

Taken together, we conclude that S130 phosphorylation by interphase CDKs is an important determinant of which substrate PHD1 will target in cells (Fig. 6F).

## DISCUSSION

In this report, we have identified and characterised the functional significance of a CDK-dependent phosphorylation site on PHD1. Our results show that phosphorylation of PHD1 on S130 is dynamic and regulated by CDK activity, cell cycle stage and oncogenic signals. Interestingly, although phosphorylation of PHD1 on S130 does not alter PHD1 hydroxylase activity *in vitro*, it regulates PHD1 activity in cells, determining target selection between HIF and Cep192. These results provide a mechanistic link between the cell cycle and the regulation of PHD1 activity in cells, allowing for its different functions to be carried out at specific stages of the cell cycle.

Although PHD2 and PHD3 are transcriptional targets of HIF1α (Metzen et al., 2005; Pescador et al., 2005), PHD1 is not induced following HIF1α activation. However, the activity of all of these enzymes is regulated by availability of cofactors, such as molecular oxygen, Fe^2+^ and α-KG (Fandrey et al., 2006; Kaelin, 2012). Little information exists about how PHD protein levels are regulated. Some studies have demonstrated that PHD3 protein turnover is regulated by the Siah-2 ubiquitin ligase (Nakayama and Ronai, 2004), whereas the FKBP38 protein, a peptidyl-prolyl cis-trans isomerase, regulates the protein stability of PHD2 (Barth et al., 2009, 2007). More recently, PHD3 has been shown to be sumoylated, which although not altering PHD3 hydroxylase activity, was important for PHD3-mediated repression of HIF1α transcriptional activity (Nunez-O'Mara et al., 2015).

Despite these studies, there was no information on how these processes are regulated and how different signalling pathways and/or cellular processes integrate with PHD function. Here, we describe how PHD1 function is regulated by phosphorylation. S130 of PHD1 is well conserved in different mammalian species and, as such, we would predict that PHD1 could be regulated in a similar manner in these species. A closely related site is also present on PHD2, where S125 has been identified as a phosphorylation site in unbiased mass spectrometry screens (Olsen et al., 2010; Zhou et al., 2013). Although no functional characterisation has been done for PHD2, these mass spectrometry screens have suggested that phosphorylation of PHD2 S125 is also regulated by the cell cycle (Olsen et al., 2010; Zhou et al., 2013). However, further research on PHD2 regulation is needed before any conclusion can be made regarding the importance of this phosphorylation site in cells under physiological conditions.

Our data suggest that interphase CDKs (CDK2, CDK4 and CDK6) are involved in the regulation of PHD1 phosphorylation on S130, but not CDK1. Previous data have indicated that CDKs show a degree of functional redundancy and can compensate for each other, with only CDK1 being essential (Diril et al., 2012). Our data supports this view. In fact, we could detect CDK2, CDK4 and CDK6 binding to PHD1, but not CDK1, further indicating that S130 is likely not a CDK1 phosphorylation site in cells. However, CDK1 was still able to phosphorylate PHD1 *in vitro*. Phosphorylation of this site was induced by serum, and by particular oncogenes, such as Myc and E2F1, both of which are known to regulate the cell cycle (Matsumura et al., 2003; Obaya et al., 2002). This strengthens the notion that phosphorylation of PHD1 at S130 could be of relevance to cancer biology and future research should be directed to investigate this site in the context of this disease.

Although PHD2 is the main regulator of HIF1α, PHD1 is also involved (Appelhoff et al., 2004). Our results show that a phospho-mimicking mutation of S130 (S130D), reduces PHD1 activity towards HIF1α, leading to increased HIF1α half-life and activity. Interestingly, this S130D mutation did not alter hydroxylase activity *in vitro*, when recombinant protein was analysed, but instead altered the interaction between PHD1 and HIF1α in cells. This could be due either to inability to bind a cofactor in cells or to a direct interference of the phosphorylation site with binding to HIF1α. Our analysis revealed that the N-terminal region of PHD1 is able to bind HIF1α without the help of the hydroxylation domain, suggesting that this latter point might be the case. The N-terminus of PHD2 has also been shown to be important for the regulation of HIF1α by PHD2, although in this case through an indirect mechanism involving a chaperone protein (Song et al., 2013). This highlights the fact that other domains of PHD enzymes can contribute to the hydroxylase activity of these enzymes in cells.

Recently, we have identified the centrosomal protein Cep192 as a target for PHD1 in cells (Moser et al., 2013). Cep192 requires a precise regulation of its expression level to allow for centrosome duplication and maturation (Joukov et al., 2014; Moser et al., 2013). As such, either too much or too little Cep192 results in a similar defect in centrosomes and causes cell cycle arrest. Given the finding presented here concerning the regulation of PHD1 function by phosphorylation, we investigated how S130 phosphorylation of PHD1 impinged on cell cycle progression and Cep192 levels. In rescue experiments, where endogenous PHD1 was depleted, exogenous expression of either wild-type PHD1 or the unphosphorylatable S130A PHD1 mutant meant that cells were able to proliferate, and both proteins restored Cep192 levels. However, the exogenous expression of the phospho-mimic mutant S130D was unable to restore proliferation and resulted in reduced levels of Cep192 when compared with control cells. In addition, we observed increased interaction between PHD1 and Cep192 in the phospho-mimic mutant S130D cells. This suggests that PHD1 at phosphorylated S130 has increased activity towards Cep192, corroborating the notion that phosphorylation of this site can alter the target specificity of PHD1. This provides a mechanism by which PHD1 function could be changed throughout the cell cycle, directing it towards specific targets in response to signals, such as high interphase CDK activity, for example.

Taken together, our results suggest a new paradigm for the regulation of PHD1 function by post-translational modifications. We have focused on characterising the functional consequences of PHD1 phosphorylation on S130 due to its conservation across species, and because of the presence of a similar site on PHD2. Despite this conservation, the effects of phosphorylation of PHD1 at S130 will probably only alter HIF levels, as the Cep192 hydroxylation site is not conserved in mice (Moser et al., 2013). However, additional post-translational modifications might also occur in these enzymes that help regulate activity and control the targeting of PHDs to specific substrates in cells. Further investigation will reveal whether this is the case.

## MATERIALS AND METHODS

### Cells

U2OS osteosarcoma cancer cells and HEK293 human embryonic kidney cells were obtained from the European Collection of Cell Cultures and grown in Dulbecco's modified Eagle medium (Lonza) supplemented with 10% fetal bovine serum (Gibco), 50 units/ml penicillin (Lonza) and 50 µg/ml streptomycin (Lonza) for no more than 30 passages at 37°C and 5% CO_2_. Stable U2OS cell lines expressing GFP, PHD1–GFP, PHD1-S130A–GFP, and PHD1-S130D–GFP were maintained with 400 µg/ml G418. U2OS-HRE-luciferase cells were maintained in 0.5 µg/ml puromycin. Cells were routinely tested for contamination.

### Plasmids

GFP-N1 was obtained from Clonetech. GFP–PHD1 was a kind gift from Eric Metzen (Essen University, Essen, Germany) and was used as a template to create the GFP-tagged PHD1-S130A and PHD1-S130D mutations by site directed mutagenesis. For the truncation mutants of PHD1, GFP–PHD1 and GFP–PHD1-S130D plasmids were used as templates. Primer sequences are available upon request.

CMV-HIF1α and CMV-Src expression constructs were obtained from Origene. E2F1 and wild-type Myc and mutant expression constructs were a kind gift from Victoria Cowling (University of Dundee, Dundee, UK). HA–CDK1 (1888, Addgene), HA–CDK4 (1876, Addgene) and HA–CDK2 (1884, Addgene) were as previously described (van den Heuvel and Harlow, 1993).

### Hypoxia induction and chemical treatments

Cells were incubated at 1% O_2_ in an *in vivo* 300 hypoxia workstation (Ruskin, UK). Cells were lysed for protein extracts and RNA extraction in the work station to avoid re-oxygenation. Whole-cell lysates were obtained using Triton lysis buffer [20 mM Tris-HCl pH 7.5, 150 mM NaCl, 1% Triton X-100, 250 mM Na_3_VO_4_, 10 mM NaF and phosphatase inhibitors 1 tablet, 10 ml (Roche cOmplete)]. RNA was extracted using Peqlab Total RNA Kit (Peqlab) as per the manufacturer's instructions.

MG132 was obtained from Merck/Millipore and used at the final concentration of 20 µM for 3 h. Desferroxamine mesylate (DFX) was obtained from Sigma and used at the final concentration of 200 µM.

### *In vitro* hydroxylation assay

Hydroxylation assays were performed as in Moser et al. (2013).

### Kinase assays

Kinases assays were performed as described in DeGregori et al. (1995). 2 µg of recombinant PHD1 protein was used per reaction.

### Antibodies

Antibodies were against the following proteins: phospho-serine-CDK substrate (2324, Cell Signaling), 1:1000; phospho-threonine (9386, Cell Signaling), 1:1000; phospho-serine (05-1000, Millipore), 1:1000; HIF1α (610958, BD Biosciences and sc-53546 Santa Cruz Biotechnology), 1:1000; HO-HIF1α (3434, Cell Signaling), 1:1000; HIF-2α (PA1-16510, Thermo Scientific), 1:1000; HIF-1β (#3718, Cell Signaling), 1:1000; β-actin (3700, Cell Signaling), 1:5000; CAIX (NB100-417, Novus Biologicals), 1:1000; HK2 (2867, Cell Signaling), 1:1000; BNIP3 (ab10433, Abcam), 1:2000; GLUT3 (53520, Anaspec), 1:1000; PHD3 (A300-327A, Bethyl labs), 1:1000; PHD1 (Bethyl A300-326A; Novus NBP1-40773), 1:1000; PHD2 (Bethyl A300-322A), 1:1000; Cep192 (Bethyl A302-324A; Novus NBP-84634), 1:500; HDAC1 (17-10199, Millipore), 1:2000; CDK1 (9116, Cell Signaling), 1:1000; CDK2 (2546, Cell Signaling), 1:1000; CDK4 (sc-260, Santa Cruz Biotechnology; 2906, Cell Signaling), 1:1000; CDK6 (sc-177, Santa Cruz Biotechnology; 3136, Cell Signaling), 1:1000; phospho-ERK1/2, 1:1000; cyclin A (sc-596, Santa Cruz Biotechnology), 1:1000, cyclin E (4129, Cell Signaling), 1:1000; E2F1 (3742, Cell Signaling), 1:1000; Src (2109, Cell Signaling), 1:1000; cMyc (gift from Victoria Cowling), 1:500; p53 (2524, Cell Signaling), 1:2000; pericentrin (28144, Abcam), 1:100; phospho-S130-PHD1 [produced in rabbits by immunisation with phospho-peptide for S130 of PHD1 (CEDGGDAPSphPSKR) and purified by Dundee Cell products], 1:200.

### Immunoprecipitation of PHD1 and sample preparation for LC-MS analysis

PHD1–GFP cells were lysed as described previously (Moser et al., 2013) and PHD1 was immunoprecipitated using GFP-TRAP^®^ magnetic beads (ChromoTek).

Immunoprecipitation eluates were separated on 1D SDS PAGE gels and stained (SimplyBlue; Invitrogen). The protein bands of interest were excised, chopped into ∼1-mm×1-mm pieces and destained at room temperature [2×30 min in 50:50, acetonitrile (ACN) and 100 mM triethylammonium bicarbonate buffer (TEAB) pH 8.5]. After 15 min dehydration in 100% ACN, proteins in the gel pieces were reduced by incubation in 25 mM tris(2-carboxyethyl)phosphine (TCEP) in 100 mM TEAB for 15 min at 37°C and alkylated by adding iodoacetamide to a final concentration of 50 mM and incubating in the dark at room temperature for 30 min.

After reduction and alkylation, the gel pieces were washed with 50:50 acetonitrile and TEAB to remove excess iodoacetamide, dehydrated in acetonitrile then dried in a vacuum to remove residual organic solvent prior to digestion. For tryptic digestion, the dried gel pieces were rehydrated using sequencing grade modified trypsin (Promega) solution (15 µl, 1 ng µl^−1^ in TEAB). Digestion was performed overnight at 37°C in 50 µl TEAB.

Digested peptides were extracted by adding 1% formic acid in acetonitrile (50 µl) to the gel pieces and incubating for 20 min at room temperature. The supernatant, now containing tryptic peptides, was transferred to a clean tube. The gel pieces were extracted further with two washes with 100 µl water:acetonitrile (50:50) incorporating 1% formic acid and once wash with 100% ACN. All extracts were combined, dried down and redissolved in 5% aqueous formic acid for liquid chromatography mass spectrometry (LC-MS) analysis.

### LC-MS analysis

The digests were analysed using a nano-LC (RSLC-Thermo Scientific) coupled to a Q-exactive orbitrap (Thermo Scientific). The peptides were loaded in 5% formic acid and resolved on a 50-cm RP-C18 EASY-Spray temperature-controlled integrated column-emitter (Thermo Scientific) using a 2-h multistep gradient of acetonitrile (5% acetonitrile to 60% acetonitrile). The chromatography was performed at a constant temperature of 40°C. The peptides eluted directly into the sampling region of the mass spectrometer, and the spray was initiated by applying 1.9 kV to the EASY-Spray (Thermo Scientific). The data were acquired under the control of Xcalibur software in a data-dependent mode, selecting the 15 most-intense ions for sequencing by tandem mass spectrometry using higher-energy collisional dissociation (HCD) fragmentation. The raw data were processed using the MaxQuant software package (version 1.3.0.5) (Cox and Mann, 2008) to identify the proteins enriched in the immuno-affinity pulldowns by searching against the human proteome database with phosphorylated serine, threonine and tyrosine as a variable modification, in addition to the commonly used post-translational modifications (protein N-terminal acetylation, methionine oxidation, asparagine and glutamine deamidation, carbamidomethyl cysteine and conversion of N-terminal glutamate into pyroglutamate).

### Additional experimental procedures

siRNA transfection, quantitative real-time PCR (qPCR), immunofluorescence, synchronisation release and cell cycle analysis experiments were performed as previously described (Kenneth et al., 2010; Melvin et al., 2011). Primer and siRNA sequences are available upon request.

### Statistical analysis

*P*-values were calculated with Student's *t*-tests (unless otherwise indicated) in all the data comparing control to treatment. *P<0.05, **P<0.01 and ***P<0.001.
